# Glyphosate and adverse pregnancy outcomes, a systematic review of observational studies

**DOI:** 10.1186/s12889-016-3153-3

**Published:** 2016-06-06

**Authors:** Jessica S. A. de Araujo, Isabella F. Delgado, Francisco J. R. Paumgartten

**Affiliations:** National Institute for Health Quality Control, Oswaldo Cruz Foundation – FIOCRUZ, Av. Brasil 4365, Rio de Janeiro, RJ 21040-900 Brazil; National School of Public Health, Oswaldo Cruz Foundation – FIOCRUZ, Av. Brasil 4036, EXCAM building, rooms 101-104, 913, Rio de Janeiro, RJ 21040-361 Brazil

**Keywords:** Attention deficit hyperactivity disorder, Pesticides, Birth defects, Congenital anomalies, Abortion, Teratogenicity, Reproductive toxicity, Occupational exposure, Agricultural workers, Fecundity

## Abstract

**Background:**

A study in frog and chicken embryos, and reports of a high incidence of birth defects in regions of intensive GM-soy planting have raised concerns on the teratogenic potential of glyphosate-based herbicides. These public concerns prompted us to conduct a systematic review of the epidemiological studies testing hypotheses of associations between glyphosate exposure and adverse pregnancy outcomes including birth defects.

**Methods:**

A systematic and comprehensive literature search was performed in MEDLINE, TOXLINE, Bireme-BVS and SCOPUS databases using different combinations of exposure and outcome terms. A case–control study on the association between pesticides and congenital malformations in areas of extensive GM soy crops in South America, and reports on the occurrence of birth defects in these regions were reviewed as well.

**Results:**

The search found ten studies testing associations between glyphosate and birth defects, abortions, pre-term deliveries, small for gestational date births, childhood diseases or altered sex ratios. Two additional studies examined changes of time-to-pregnancy in glyphosate-exposed populations. Except for an excess of Attention Deficit Hyperactivity Disorder - ADHD (OR = 3.6, 1.3-9.6) among children born to glyphosate appliers, no significant associations between this herbicide and adverse pregnancy outcomes were described. Evidence that in South American regions of intensive GM-soy planting incidence of birth defects is high remains elusive.

**Conclusions:**

Current epidemiological evidence, albeit limited to a few studies using non-quantitative and indirect estimates and dichotomous analysis of exposures, does not lend support to public concerns that glyphosate-based pesticides might pose developmental risks to the unborn child. Nonetheless, owing to methodological limitations of existing analytical observational studies, and particularly to a lack of a direct measurement (urine and/or blood levels), or an indirect estimation of exposure that has proven valid, these negative findings cannot be taken as definitive evidence that GLY, at current levels of occupational and environmental exposures, brings no risk for human development and reproduction.

## Background

Glyphosate (*N*-(phosphonomethyl)glycine, GLY) is a broad-spectrum herbicide launched onto the market in 1974. It inhibits competitively the enzyme enolpyruvylshikimate phosphate synthase (EPSPS) thereby blocking the production of chorismate, an intermediate for the biosynthesis of phenylalanine, tyrosine and tryptophan [1]. Contrasting to plants, bacteria and fungi, animals do not have the genes coding for EPSPS and depend on a dietary source for these essential aromatic amino acids. Although exhibiting plant- selective toxicity, GLY-based herbicides might eventually impair human health through modes of action other than inhibition of a shikimate metabolic pathway [1].

Pre-marketing safety evaluation of GLY and its major breakdown product AMPA (aminomethylphosphonic acid) did not reveal substance-related significant health hazards. Some post-marketing toxicity studies conducted by academic researchers, however, raised concerns about possible adverse health consequences of occupational exposures to GLY-based herbicides [2–4]. A methodological constraint common to many of these non-industry toxicity studies is the use of a commercial product (e.g., Roundup®) rather than the active ingredient (GLY), and the lack of adequate controls for the effects of formulation ingredients other than GLY. Formulated GLY-based herbicides often contain substantial amounts of surfactants (e.g., polyoxyethyleneamine or POEA) the biological effects of which may account for product toxicity.

Since the introduction of GLY-resistant genetically modified (GM) soybean in the 1990s, GLY has become the most extensively used herbicide in Brazil, USA and other major soybean production countries. Moreover, the ADI (Acceptable Daily Intake) for GLY established by FAO/WHO augmented from 0–0.3 mg/kg bw/d (1986) to 0–1.0 mg/kg bw/d (2004), while maximum residue concentrations (MRCs) for GLY in soy and several other food crops increased as well [5]. A recent classification of GLY as “probably carcinogenic to humans” (group 2A) by the IARC fueled the ongoing debate about the safety of this herbicide. Classification of GLY into 2A group was based on “limited” evidence in humans (association with non-Hodgkin lymphomas) and “sufficient” evidence of carcinogenicity in animals [6]. Because of IARC classification and growing public concerns on the safety of GLY, the Brazilian regulatory agency (ANVISA) has scheduled a re-evaluation of GLY-based herbicides for 2016.

Whether the extensive use of GLY poses a risk to human reproductive health is a controversial matter as well. Studies sponsored by pesticide manufacturers revealed no noticeable adverse effects of GLY on mammalian reproduction and prenatal development [1]. Nonetheless, in 2010, Paganelli et al. [4] described a harmful effect of GLY-based herbicides on the development of frog (*Xenopus laevis*) and chicken embryos. According to the authors, the neural crest was the primary target of GLY and the mode by which GLY disrupts embryo development would be similar to that of retinoic acid, a known potent human teratogen [4, 7]. Along the same line, two experimental studies performed by independent researchers suggested that GLY might impair pre and postnatal development in rats [2, 3]. Dallegrave et al. [2, 3] reported embryotoxic effects (delayed skeleton ossification in term fetuses) in the offspring of rats treated with a GLY-based herbicide during pregnancy, and a decreased sperm count in adult rats that had been exposed in utero and during lactation.

South America rural regions are particularly suitable for studies of GLY impact on human health. In Brazil, Paraguay and Argentina, there are large GM-soy crop areas and extensive use of GLY, while in Colombia and Equator attempts to eradicate illegal crops of coca and poppy involved aerial spraying of GLY-based herbicides [8]. A suspected increase in the incidence of birth defects in South American GM soy-crop areas has been cited in support to the notion that GLY might be a human developmental toxicant [4, 7, 9–11]. A putative teratogenicity of GLY has been often commented in the literature. Samsel and Seneff [12], for instance, speculated that GLY might play a role in the pathogenesis of celiac (and gluten intolerance) disease and proposed that adverse influence of maternal celiac disease and GLY on prenatal development might share a common mode of action.

This systematic review was undertaken to evaluate whether results from the existing epidemiological studies are consistent with the notion that maternal and/or paternal exposure to GLY increases the risk of birth defects and/or other adverse pregnancy outcomes.

## Methods

### Literature sources and search strategy

A search was conducted in MEDLINE, TOXLINE, BIREME/BVS (Health Virtual Library-Brasil / “Biblioteca Virtual em Saúde–Brasil”) and SCOPUS electronic databases to identify studies on human reproductive and/or developmental outcomes and GLY-based pesticide exposures. The literature search covered time windows between the inception of the database and May 9th, 2015 (MEDLINE), May 12th, 2015 (BIREME/BVS), May 14th, 2015 (TOXLINE), and May 15th, 2015 (SCOPUS). The search in BIREME/BVS database covered articles published in periodicals indexed in the Latin American and the Caribbean Literature on Health Sciences or LILACS (“*Literatura Latino-Americana e do Caribe em Ciências da Saúde*”). LILACS, the most comprehensive index of scientific and technical literature on health of Latin America and the Caribbean, includes local journals not indexed in the other databases searched in this study. There was no restriction regarding the language of the article.

The search in all four databases combined a specific exposure term and one term related to the health outcomes of interest in English (i.e., “glyphosate AND developmental toxicity”, “glyphosate AND birth defects”, “glyphosate AND congenital anomalies”, “glyphosate AND embryotoxicity”, “glyphosate AND epidemiology”, “glyphosate AND pregnancy”, “glyphosate AND teratogenicity”, “glyphosate AND reproductive outcomes”, “glyphosate and abortions”, “glyphosate AND malformations”, glyphosate AND pregnancy outcomes”, “Round up AND developmental toxicity”, “Round up AND birth defects”, “Round up AND congenital anomalies”, “Round up AND embryotoxicity”, “Round up AND epidemiology”, “Round up AND pregnancy”, “Round up AND teratogenicity”, “Round up AND reproductive outcomes”, “Round up and abortions”, “Round up AND malformations”, “Round up AND pregnancy outcomes”. To ensure the completeness of the search, the reviewers cross checked reference lists in the selected articles and reviews on this topic to identify by hand searches any relevant studies that might have been eventually missed by the electronic search.

### Study selection and data extraction

To be included in the review an observational study had to investigate whether paternal and/or maternal exposures to GLY before conception and/or during pregnancy increased risks of adverse pregnancy outcomes, childhood health disorders and/or changes in fecundability. Pre-determined study criteria for exclusion were as follows: 1) in vitro experimental studies, 2) in vivo and *ex vivo* studies in animals, 2) studies of effects on non-target species other than humans, 3) studies in the area of pesticide chemistry and agricultural sciences, 4) report of clinical cases, series of cases, and treatment of intoxications, 5) studies of associations of postnatal exposures with non-developmental and/or non-reproductive health outcomes, 6) letters, reviews, editorials, reports, comments, documents issued by regulatory bodies, and book chapters.

The selection of studies for reviewing involved a two-phase screening process: at first publications were screened by titles and abstracts, and if deemed potentially relevant, at a subsequent phase, full text articles were retrieved. Screening of records for study eligibility was conducted independently by two reviewers (JAA and FJRP). If a disagreement persisted after being extensively discussed by the two reviewers, a third investigator (IFD) was asked to resolve it. For the qualitative synthesis the following data were extracted from the selected articles: study type, the studied population, its size, location and time, how exposure to GLY was assessed, the adverse outcome of pregnancy and/or the reproductive outcome evaluated, whether or not the outcome was significantly associated with GLY, and the strength of association (e.g. OR) if available.

## Results

**Fig. 1:**
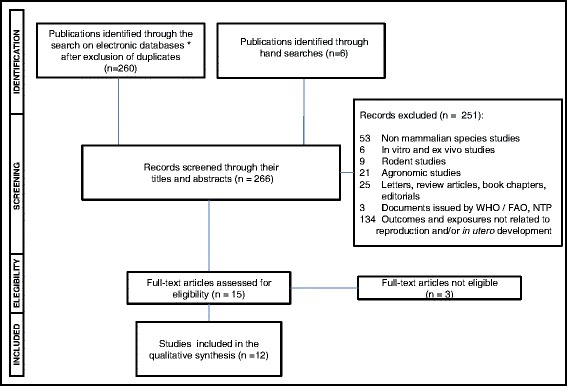
Flowchart of selection of observational studies for inclusion in the systematic review. *MEDLINE, TOXLINE, Bireme/BVS and SCOPUS

After excluding all duplicates, the total number of documents retrieved by our search on multiple electronic databases was 260. Six additional articles potentially eligible were identified through hand-searches. Of these 266 records, 251 documents were excluded by their titles and abstracts. Most epidemiologic studies found by the search in electronic databases investigated whether paternal and/or maternal exposure to multiple pesticides (including GLY) was associated with a higher risk of decreased fecundity, miscarriages and congenital malformations and/or functional disorders in the progeny. Only the studies testing whether GLY-based herbicides were associated with altered fecundity and/or adverse pregnancy outcomes were eligible for review. As shown in the flowchart depicted in Fig. 1, 3 out of 15 full-text articles assessed for eligibility were excluded and the reasons for their ineligibility are informed in Table 1. Seven out of the 12 remaining studies addressed the question as to whether parental exposures to GLY increased risks of birth defects and/or childhood functional disorders in the offspring (Table 2), while the other five studies investigated potential effects of exposure to this herbicide on time-to-pregnancy, pre-term deliveries, small-for-gestational-age births and pregnancy losses (Table 3).

**Table 1:** Characteristics of excluded studies (full text articles) and the reason for their ineligibility

Study	Study type, population and location	Exposure assessment	Conclusions	Reason for ineligibility
Pogoda and Preston-Martin, 1997. [35]	Follow up to population-based case–control study of pediatric brain tumors in Los Angeles county, CA, US, from 1984 through June 1991. Cases, *N* = 224, Controls, *N* = 218.	Household pesticide use from pregnancy to diagnosis assessed by phone interviews.	Risk was elevated for flea-tick pesticides, OR = 1.7, 1.1-2.6.	Round up was listed among pesticides (herbicides) used in law-garden. However, only associations between broad classes of pesticides and the outcome (brain tumors) were tested. Exposure assessment encompassed both pre and postnatal development and did not discriminate between the two periods.
Larsen et al., 1998. [36]	Retrospective study of time-to-pregnancy (fecundability) among Danish farmers (*N* = 1146). Telephone interviews on time-to-pregnancy (How many months did it take your wife to get pregnant?) and reproductive history	Exposure data obtained by phone interviews (use of pesticides the year before the youngest child was born). Exposed groups: traditional farmers, traditional farmers who did not spray pesticides themselves, organic farmers.	Fecundability ratio between “traditional farmers who applied pesticides” and “organic farmers”: 1.3, 0.75 – 1.40.	Although GLY is mentioned in the Introduction section, authors did not investigate whether ‘time-to-pregnancy” was altered specifically by this herbicide. The authors tested only classes of “pesticides”.
Nevison 2014. [37]	Ecological-type study design. Temporal trends (1970–2005) in autism compared to temporal trends in US application of GLY. Autism counts were from the Individuals with Disabilities Education Act (IDEA) database for 50 US states plus District of Columbia.	GLY exposure data were aggregated at the group level. Data were from US department of agriculture.	Increasing trends in application of GLY positively correlated to a marked rise in autism prevalence in the US.	In this study of temporal trends, data on potential exposure were aggregated at the whole US population level and encompasses preconception, pre-natal and postnatal periods of development. Moreover, the ecological study design leaves the door wide open for non-causal explanations for the observed parallel rise in temporal trends in GLY use and autism prevalence.

*GLY* glyphosate

**Table 2:** Studies of parental exposures to glyphosate and birth defects or functional disorders in the progeny

Study	Study type, population and location	Exposure assessment	Adverse pregnancy outcome	Association with GLY – Statistical Analysis OR (CI 95 %)
Garcia et al., 1998 [13]	Case–control study with 261 matched pairs in 8 hospitals of Comunidad Valenciana, Spain.	Paternal exposure. Fathers were interviewed for obtaining detailed information on direct involvement in handling of pesticides.	Congenial malformation (any)	1.23 (0.59-2.56) crude
				0.94 (0.37-2.34) adjusted
Rull et al., 2006 [14]	Case–control study. Rural California US population (California Birth Defects Monitoring Program) -1987-1991 (pooled data of 2 case–control studies)^b^. Controls: unmatched randomly sampled from all live born in the same sampling time without congenital anomalies diagnosed before the 1st birthday.	Maternal residential proximity (<1,000 m) to pesticide sprayed crops during early pregnancy /questionnaire (residential addresses 2 weeks or more during the periconceptional period linked to geographic distribution of crops; pesticide use reports).	NTD^a^	Conventional logistic regression models
				**1.5** (**1.0**-**2.4**)-Single pesticide
				1.5 (0.8-2.9)-Multiple pesticides
Yang et al., 2014 [16]	Case–control study. CA Birth Defects Monitoring Program, 1997–2006. San Joaquin Valley CA US. Cases (*n* = 763): infants/fetuses with anencephaly or spina bifida and those with cleft palate (CP) or cleft lip (CL) with or without CP. Diagnoses confirmed by clinical geneticists. Cases suspected to having singe-gene conditions, chromosome abnormalities and an identified syndrome were not eligible. Controls (*n* = 974) non-malformed live-born infants selected at random from birth hospitals.	Maternal residential proximity to pesticide sprayed crops during periconceptional period (time window of exposure: 1 month before to 2 monthsnths after reported date of conception) (computer-based questionnaire administered primarily by telephone 6 weeks to 24 months after the date of infant delivery). Mothers with diabetes were excluded.	NTD, orofacial clefts	0.9 (0.5-1.9) – anencephaly
				0.9 (0.5-1.4) – spina bifida
				0.9 (0.7-1.3) - CL w/wo CP
				0.9 (0.5-1.5) – CP alone
Carmichael et al., 2013 [15]	Case–control study. Study population was all male infants born from 1991–2004 to mothers who were residents of 8 CA Central Valley counties. Cases (*n* = 690) were infants with hypospadias ascertained by the CA Birth Defects Monitoring Program (1991–2004); Controls (*n* = 2195) were live-born male infants with no major malformation selected randomly from the birth population (*n* = 2195).	Maternal residential proximity (within a 500 m radius) to pesticide sprayed crops during early pregnancy (1–98 days after reported date of conception; 1–14 weeks embryonic age) /questionnaire. Birth certificate accession numbers were used to request access to maternal residential addresses at delivery. Data on pesticide applications (Pesticide Use Reporting records) were from the CA department of pesticide regulation.	Hypospadias	0.68 (0.34-1.37) – lowest (total amount applied <1.22 lb)
				0.44 (0.19-1.01) – intermediate (total-amount applied 1.22-6.92 lb).
				0.88 (0.48-1.64) – highest (total amount applied ≥6.93 lb).
Shaw et al., 2014 [17]	Case–control study. Study population was from the San Joaquim Valley of California, US (1997–2006). Cases were 156 infants/fetuses were infants with gastroschisis confirmed by clinical geneticists and the controls (*n* = 785) were non-malformed live-born infants randomly selected from birth hospitals to represent the population from which the cases arose. Cases with recognized or suspected to have single-gene conditions or chromosomal abnormalities or with identifiable syndromes were ineligible. Cases and controls whose mothers had diabetes were excluded from analysis.	Residential proximity (within 500 m radius) to pesticide applications (to estimate pesticide applications data were obtained from Pesticide Reporting Records from the California Dept of Pesticide Regulation). Maternal interviews (by phone) using a standardized computer-based questionnaire took place between 6 weeks and 24 months after infant’s estimated date of delivery. Mothers reported residential history from 3 month before conception through delivery, including dates and residences occupied for >1 month. Time window of exposure analyzed went from 1 month before to 2 months after the mother reported date of conception.	Gastroschisis	0.9 (0.6-1.4) – crude OR
				0.9 (0.6-1.5) – adjusted OR
Garry et al., 2002 [18]	Cross-sectional study. Certified pesticide appliers (licensed between 1991 and 1996) in Red River Valley, Minnesota US. Participants randomly selected were invited by phone interview and asked to return a detailed written questionnaire on reproductive health and pesticide use assessment. Informants were at least one member of 695 families (228 male spouse applicators, 90 female spouses, 377 couples).	Each certified pesticide applicator was interviewed by phone on current and past use of pesticides with attention to product name, years used and no. of days per year applied. Approximately 6 months later participants were interviewed again by a written questionnaire. Spouses were also interviewed by phone and again by written questionnaire. Frequencies of the outcome were compared between those who reported the use of GLY and those who did not report the use. (dichotomous pesticide exposure).	ADD/ADHD	**3.6** (**1.3**-**9.6**)
Waselak et al. 2007 [19]	Cross-sectional study. Farm couples were from the Ontario Farm Family Health Study (OFFHS), a study designed to retrospectively assess the effects of pesticide exposures on reproductive health. Couples were eligible if they were married or living as married, living year-around on a farm operation, and the wife was at most 44 years of age. Questionnaire were mailed to each farm family to get information on health status, pesticide use and farm-activity exposures. Farm wives were asked to self-report if “the doctor has ever told them” if their child had had chronic bronchitis or cough, asthma, hayfever or allergies.	Information on agricultural chemicals used on the farm 6 largest crops sown or harvested in 1991 were obtained through a questionnaire addressed to the farm operator. Information on historical farm chemical used was obtained by questionnaire. Questionnaires addressed to husband and wife asked information pertaining to chemical activities on the farm and around the home. The year and month of chemical use was matched with months and years leading up to and of each pregnancy. Pesticide exposures during the pregnancy period (month of conception until the month of delivery) were considered in the analysis of child health outcomes. Unexposed pregnancies were those with no reported pesticide use during the same period. Analysis: ratios exposed cases to total exposed to GLY.	Persistent cough / bronchitis, asthma, allergies	Male and female offspring combined (OR adjusted): cough/ bronchitis 0.71 (021–2.35) asthma 0.82 (0.35-1.90) hayfever/ allergies 0.98 (0.46-2.10)

*GLY* glyphosate, *SGA* Small for Gestational Age, *CL* cleft lip, *CP* cleft palate, *NTD* neural tube defect, *TTP* (Time-to-pregnancy) is defined as the duration that a couple waits from initiating attempts to conceive until conception occurs. TTP is used to calculate probability of conception or the fecundability odds ratio (fOR). NTD ^a^ including elective termination of pregnancy, confirmed diagnosis of anencephaly, spina bifida cystica, craniorrhachischisis and iniencephaly. First study: children of women in most CA counties, Second study: all CA counties except LA, Ventura and Riverside. *ADD/ADHD* Attention Deficit Disorder/Attention Deficit Hyperactivity Disorder. Significant associations are highlighted marked in bold type

**Table 3:** Studies of parental exposures to glyphosate and reproductive outcomes other than birth defects

Study	Study type, population and location	Exposure assessment	Reproductive outcome	Association with GLY – Statistical Analysis OR (CI 95 %) / CFR^a^
Savitz et al., 1997 [20]	Retrospective cohort study. Ontario Farm Family Health Study (OFFHS), farms likely to be full-time family-run operations were selected based on 1986 Canadian Census of Agriculture. Couples were considered as eligible (telephone interview) based on residence on or near the farm-year-round, and age of female partner (≤44 years old). Among 1898 farm couples with completed questionnaires (from farm operator, husband and wife), 3984 pregnancies were identified.	Paternal exposure assessed by questionnaire interview. Men were asked about their farm activities over the past 5 years. Five activities were presumed to involve direct pesticide exposure: mixing or applying crop herbicides, insecticides, and fungicides, yard herbicides, livestock chemicals and building pesticides. Male partners engaged in any activity associated with direct pesticide exposure for ≥1 month in the time window of 3 months before conception to the time of conception.	Abortion	1.5 (0.8-2.7) – adjusted– abortion (crop)
				1.4 (0.7-2.8)–adjusted– abortion – (yard)
				2.4 (0.8-7.9)–adjusted– pre-term delivery (crop)
				0.8 (0.2-2.3)–adjusted- SGA- (crop)
			Pre-term delivery	
			SGA births	
Curtis et al., 1999 [21]	Retrospective cohort study. OFFHS – same as described above. Only planned pregnancies were selected for the analysis regardless of the pregnancy outcome.	Paternal exposure assessed by questionnaire interview. Pesticide used on the farm (monthly use) during the month trying to conceive or at any time during the prior 2 months to capture the time dependent exposure interval that may have affected spermatogenesis (64 days).	Fecundability	GLY exposure / CFR^a^ : adjusted
				Woman activity regardless man activity - 0.61 (0.30-1.26)
				Man activity / no woman activity **1.30** (**1.07** – **1.56**) (**↑fecundability**) Farm pesticide use/no couple activity
				1.26(0.94-1.69) No pesticide use/engaged in activities 1.00 (0.91-1.09)
Arbuckle et al., 2001 [22]	Retrospective cohort study. OFFHS – same as described above	Paternal exposure assessed by questionnaire interview. Male farm activities in the period from 3 months prior to conception through the month of conception were assessed by questionnaire and evaluated in relation to miscarriages, pre-term delivery and SGA births.	Abortion	**1.7 (1.0-2.9)**–pre-conceptional exposure and late abortion (12–19 weeks) 1.1 (0.7-1.9) – pre-conceptional exposure and early abortion (<12 weeks) 1.1 (0.7-1.7) – post-conceptional exposure and abortion at any gestational age.
Sanin et al., 2009 [23]	Ecological-type study design. 2004–2005 Outcome was time to pregnancy evaluated by questionnaire interview (*How many months have you having sexual intercourse before you became pregnant for the first time?*) women were invited to participate if their first pregnancy during the last 5 years and did not take contraceptives the year before becoming pregnant. 2751 women were included in the analysis. 159 women who reported consultation with a physician for fertility problems were excluded from multiple regression and alternative models.	Exposure measurement: Five regions of Colombia with different agricultural practices and presence or not of aerial spray for eradication of illicit crops with GLY	Time-to-pregnancy	Aerial spraying of GLY was not consistently associated with a delayed time to pregnancy.
Sathyanarayana et al. 2010 [24]	Multiple regression analysis of birth weight. Pesticide applicators and their spouses enrolled between 1993 and 1997, Iowa and North Caroline US. (2246 women).	Exposure assessed by interview and questionnaire	Birth weight	Mean birth weight ± SD: 3586 ± 546 g.
				GLY-associated change in birth weight: 4 g, −40 to + 48 g. (NS)

*CFR* ^a^ Conditional fecundability ratio, *NS* non-significant, *GLY* glyphosate, *SGA* small for the gestational age. Significant associations are highlighted in bold type. ↑fecundability = increase in fecundability

**Table 4:** Incidence of congenital malformations in the Chaco province (La Leonesa-Las Palmas) between 1997 and 2009

Year (12 mo)	Malformed infants (hospital-based records, N)^a^	Births in the region (population-based registry) (N)	Birth defects rates (/ 10,000 births)
1997-8	46	24,030	19.1
2001-2	60	21,339	28.1
2008-9	186	21,808	85.3

^a^Neonatology Department, Hospital Perrando, Resistencia, Chaco Province, Argentina. Data were from the report by the Water Pollutants Investigation Committee, Chaco Province, Argentina, 2010 [27]

### Birth defects and childhood diseases in the progeny

A case–control study by Garcia et al. [13] investigated whether father exposure to pesticides increased the risk of birth defects. Garcia et al’s study considered only congenital anomalies or groups of defects with a high prevalence at birth. Controls were non-malformed live-born babies matched (1:1) with the cases by hospital and date of birth. Individual exposures were assessed by interviewing (questionnaire) fathers and mothers for involvement in activities potentially related to pesticide exposure. The risk period for exposure was defined as 3 months before conception or during the 1st trimester or both for the father and during the 1st month preceding conception and the 1st trimester or both for the mother. Risk estimates were calculated for all birth defects jointly. Dichotomous (exposed/ not exposed) data analysis revealed a positive association between exposure to pyridil derivatives and malformations (adjusted OR, 95 % CI, 2.77, 1.19-6.44), whereas no association between GLY (0.94, 0.37-2.34) and other pesticides with congenital anomalies was detected.

Rull et al. [14] pooled data from two case–control study populations of infants with Neural Tube Closure Defects (NTD) and non-malformed controls (California, US, 1987–1991) to investigate whether maternal residential proximity (within 1000 m) to pesticide application areas during early pregnancy would increase the risk of these birth defects. Results revealed a significant association between residential proximity to GLY application sites and NTD in the offspring when a single-pesticide model of conventional logistic regression analysis was used (OR = 1.5; 1.0-2.4). The association of GLY with NTD, however, was not significant when a multiple-pesticide model was adopted in the logistic regression analysis (OR = 1.5; 0.8-2.9).

Carmichael et al. [15] conducted a case–control study to evaluate whether maternal residential proximity (within 500 m radius) to agricultural pesticide application areas during early pregnancy (i.e., 1–14 weeks of embryonic age or 1–98 days of date of conception) was associated with an increased incidence of hypospadias in the offspring (data collected by the investigators refer to births occurring from 1991 to 2004 in 8 counties in CA, US). Carmichael et al’s study found no association of most pesticides including GLY with increased risks of hypospadias (phosphonoglycine, highest dose group: OR = 0.88, 0.48-1.64).

Yang et al. [16] investigated whether maternal exposure to pesticides (including GLY) during periconceptional period (i.e., from 1 month before to 2 months after the estimated date of conception) increases the risks of NTDs and/or orofacial clefts in the progeny. Maternal exposure to pesticides was inferred from the residential proximity to sprayed crops assessed by a questionnaire. Case–control analysis (based on data from the US National Birth Defects Prevention Study, from 1997 to 2002) found no association between GLY (phosphonoglycine) and anencephaly (OR = 0.9, 0.5-1.9), spina bifida (OR = 0.9, 0.5-1.4), cleft lip with and without cleft palate (OR = 0.9, 0.7-1.3) and cleft palate alone (OR = 0.9, 0.5-1.5).

Shaw et al. [17] conducted a case control study in a population derived from one of the regions of highest use of pesticide in the US (San Joaquin Valley – CA) to examine whether early gestational exposures to pesticides (including GLY) were associated with risk of gastroschisis. Exposure during early gestation was assessed by residential proximity (within 500 m radius) to GLY/pesticide applications from one month before to 2 months after mother reported date of conception. The investigators found no association between gastroschisis and maternal exposure to GLY (adjusted OR = 0.9 (0.6-1.5).

In addition to the foregoing studies on GLY and congenital malformations, three other studies investigated associations of prenatal exposures to GLY with postnatal functional disorders in the exposed offspring.

A cross-sectional study by Garry et al. [18] conducted in 1997–1998 examined the incidence of birth defects and sex ratios in children born to pesticide applicators (695 families) living in the Red River Valley of Minnesota, US. The investigators employed a detailed questionnaire to assess parental exposure to pesticides and examined medical records and birth certificates to confirm parent-reported health information. Of 19 children with Attention Deficit Hyperactivity Disorder (ADD/ADHD), 5 were excluded from the analysis because the male applicator was reported as not being the biological father. The investigators found that 6 out of the remaining 14 children (43 %) who had parent-reported ADD/ADHD were born to GLY or phosphonamine herbicide applicators (OR = 3.6, 95 %-CI, 1.35-9.65).

Weselak et al [19] used data from the Ontario Farm Family Health Study (OFFHS) to investigate whether exposure of farm couples to pesticides (including GLY) during pregnancy increases the risk of health problems in their offspring, including persistent cough or bronchitis, asthma, and allergies or hay fever. The authors found no association between GLY exposure during pregnancy and persistent cough or bronchitis (male and female offspring combined, adjusted OR, CI 95 %, 0.71, 0.21-2.35), asthma (0.82, 0.35-1.90), and hay fever and allergies (0.98, 0.46-2.10).

In summary, except for positive associations of parental exposure to GLY with ADD/ADHD [18] and NTD (single-pesticide model of logistic regression) [14], we found no other evidence suggesting that parental exposure to GLY before conception and/or during pregnancy increases the risk of birth defects and/or psychiatric or immune system disorders in the progeny.

### Miscarriages, pre-term deliveries, small-for-gestational-date births, and fecundability

Three retrospective cohort studies based on data from OFFHS study were published in the late 1990s and early 2000s. The OFFHS study was designed to assess retrospectively the potential adverse effects of commonly used pesticides on pregnancy (Table 3).

Savitz et al [20] examined the effects of male partner self-reported exposure to pesticides (0 to 3 months prior to conception) on female partner miscarriages, pre-term deliveries and small for gestational age births (SGA). Among 1898 farm couples with completed questionnaires, 3984 pregnancies were identified. Of the included pregnancies, nearly 40 % occurred 10 or more years before the interview, a long time interval that possibly enhanced the recall bias. Adjusted OR (CI 95 %) for GLY and miscarriages was 1.5 (0.8–2.7) and 1.4 (0.7–2.8) for crop and yard herbicides, respectively. No significant associations were found for GLY and pre-term deliveries (2.4, 0.8-7.9, crop), and GLY and SGA (0.8, 0.2-2.3, crop).

A study by Curtis et al [21], including only planned pregnancies (*N* = 2012), evaluated whether exposure to pesticides, assessed considering farm use and individual farm activities, was associated with a longer time-to-pregnancy. The results indicated that the use of GLY on the farm and pesticide activities reported by the husband, but not by the wife, was associated with a slightly increased fecundability (Conditional fecundability ratio; CFR, CI 95 %, 1.30, 1.07-1.56). The authors found no other significant association between GLY and altered time-to-pregnancy. Overall, study results are consistent with the notion that exposure to GLY in farm activities did not impair fecundability.

A third study based on OFFHS data [22] found a significant association (OR = 1.7, 1.0-2.9) between parental pre-conception exposure to GLY and the risk of late abortions (12–19 weeks). The risk of early abortions, however, remained unaltered (<12 weeks; OR = 1.1, 0.7-1.9) if parents were exposed to GLY prior to conception. Post-conception exposure to GLY did not increase the risk of abortion at any gestational age (OR = 1.1, 0.7-1.7). A positive association between exposure to GLY before conception and late abortions is an unusual finding. Maternal and/or paternal preconception exposures are generally associated with early abortions (e.g., due to germ cell chromosomal abnormalities) while maternal exposures during pregnancy are most often associated with late abortions (e.g., due to harmful effects on the conceptuses).

An ecological-type study by Sanin et al [23] examined whether application of glyphosate by aerial spray for eradication of illicit crops (five Colombian departments, cross-sectional study, 2004–2005) was associated with prolonged time-to-pregnancy among fertile women living in sprayed areas. While data on the exposure to GLY were aggregated at the group level and place of residence is an inaccurate surrogate for degree of exposure, the study by Sanin et al (2009) showed no consistent association between aerial spray of GLY and longer average time-to-pregnancy in the population (Table 3).

Sathyanarayana et al [24] examined the association between maternal pesticide use and birth weight among women in the Agricultural Health Study (AHS), a large study of pesticide applicators and their spouses, enrolled between 1993 and 1997 in Iowa and North Caroline. The authors evaluated self-reported pesticide use of 27 active ingredients in relation to birth weight among 2246 women. Multiple regression estimates of change in birth weight (adjusted for site, maternal BMI, age, smoking, race, pre-term birth, medical parity) were obtained. Sathyanarayana et al found no association between birth weight and parental exposure to GLY (mean birth weight = 3586 ± 546 g; GLY-associated change in birth weight, 4 g; −40 to 48) (Table 3).

In summary, except for a positive association between parental pre-conception exposure to GLY and the risk of late abortions [22], studies included in this review revealed no evidence of increased risks of miscarriages, pre-term deliveries, small-for-gestational age births and impaired fecundability associated with occupational exposures to GLY-based herbicides before conception and/or during pregnancy.

### Reports on the occurrence of birth defects in South America GM soybean crop areas

Antoniou et al (2012) and others [9–11] cited two studies by South American researchers [24, 25] and one report from Chaco province (Argentina) health authorities in support of their statement that regulatory agencies have neglected the existing evidence suggesting that GLY might increase the risk of birth defects. The two studies were published in Spanish in South American medical journals [25, 26], while an English version of Chaco province report is available on the web [27]. None of these studies were identified by our search in electronic databases.

A case–control study by Benítez-Leite et al [25] found an increased rate of birth defects in neonates born to women exposed to unspecified pesticides during pregnancy. Although the article did not mention explicitly GLY-based herbicides, cases and controls came from a regional hospital in Itapúa Department (Paraguay) where GLY is extensively used in GM-soy crops. Conclusions from Benitez-Leite et al’s investigation should be interpreted with caution due to study methodological weaknesses including 1) the lack of adequate control for selection and/or confounding bias, i.e., cases and controls were not matched according to a number of variables of interest (e.g., maternal age, use of medicines and others), nor were logistic regression and stratification used in the analysis of data; 2) an uncontrolled recall bias (mothers of malformed and healthy infants are known to remember differently past exposures); 3) the lack of detailed information on the applied questionnaire and on how interviewers were trained to avoid introducing an information bias (the interviewers were hospital internal doctors and nurses who were aware of the case or control status of the respondent); and 4) the inaccurate and dichotomous (Yes/No) assessment of pesticide exposure.

A cross-sectional study by Campaña et al. [26] reported the prevalence of 27 congenital anomalies in 7 geographic regions of Argentina including Cordoba and other provinces of intensive GM-soy planting and heavy use of GLY. The estimated prevalence of 27 birth defects in distinct regions was based on a sample of 21,844 malformed infants selected from a total of 855,220 births in 59 Argentinean hospitals of the ECLAMC (Latin American Collaborative Study on Congenital Malformations) network between 1997 and 2007. The estimated frequencies for 14 birth defects were higher in one or more regions when compared to the overall incidence for all 7 regions combined. For instance, the prevalence of spina bifida in Cordoba (CEN region, 12.2, 10.1-14.3) was somewhat higher than the overall prevalence in all regions combined (TOTAL, 9.9, 9.2-10.6). It was similar, however, to the prevalence of spina bifida in Buenos Aires metropolitan region (MET, 13.2, 11.8-14.7). The prevalence of anencephaly in Cordoba (CEN, 7.9, 6.3-9.8), on the other side, was similar to the prevalence estimated for all regions combined (TOTAL, 7.5, 6.9-8.1) and somewhat lower than the prevalence in Buenos Aires region (MET 10.3, 9.1-11.7). Overall, differences in the estimated prevalence of birth defects among regions were not consistent with the interpretation that there is an increased prevalence of NTDs and other congenital anomalies in Cordoba and/or in other regions of extensive use of GLY-based herbicides. It is of note that ECLAMC is a hospital-based (and not a population based) monitoring system, and thus regional frequencies estimated by Campaña et al. [26] do not necessarily reflect the prevalence of a birth defect in the population of the region. In addition to the foregoing, even if population based frequencies of some anomalies vary among regions, there exist many other possible explanations for inter-region differences including secular trends, altitude above sea level, folic acid fortification of wheat flour (introduced in Argentina in 2005, i.e., within the time period of study data gathering: 1997–2007), ethnicity, maternal age (not adjusted by authors’ analysis) and a diversity of environmental factors and exposures.

An Investigation Committee commissioned by the government of Chaco province (Argentina) described a nearly three-fold increase in the incidence of birth defects in the region of La Leonese-Las Palmas during the last decade [27, 28]. Since the apparent rise in the incidence of congenital anomalies paralleled a marked GM-soy crop expansion, the extensive use of GLY in the region has emerged as a suspected determinant factor [27, 28]. To estimate regional incidence rates, the Chaco province investigation committee divided the number of malformed newborns recorded by a single regional hospital (i.e., a hospital-based registry) by the overall number of births in the region (a population-based registry) for a given period of time (Table 4). While the overall number of births in the region remained fairly constant across the time period analysed (1997–2009), the number of malformed newborns recorded by the regional reference hospital dramatically increased in 2008/2009. Nonetheless, it is unclear whether the incidence of birth defects did in fact increase in the region since a hospital-based registry is not a reliable surrogate for a population-based registry of birth defects. Not only a number of births in the region may have occurred outside the regional hospital, but also the hospital may have received an undetermined number of pregnant women living outside the region of interest.

In summary, the Chaco province report [27], the case–control study by Benitez-Leite et al. [25] and the Campaña et al. [26] descriptive study do not lend support to the interpretation that the incidence of birth defects has increased in areas of extensive GM-soy plantations and GLY application.

## Discussion

As previously commented, this review included only studies that provided results specific for GLY exposure and reproductive and developmental outcomes, that is, studies analyzing only whole classes of compounds such as pesticides and herbicides or other broad categories of active ingredients were ineligible for inclusion in the review.

This systematic review identified 7 observational studies addressing the question of whether pre-conception (paternal and/or maternal) and/or pregnancy (maternal) exposures to GLY increase risks of birth defects and/or childhood functional disorders in the offspring (Table 2). Moreover, we also reviewed 5 studies evaluating whether parental exposures to GLY increased risks of miscarriages, pre-term deliveries, SGA births and prolonged time-to-pregnancy (Table 3). None of the studies found significant associations of parental exposures to GLY with unspecified congenital malformations [13] or with specific birth defects such as gastrochisis [17], oral clefts [16] and hypospadias [15]. One study found a positive association of maternal residential proximity to GLY application sites with NTDs (with a single-pesticide model but not with a multiple pesticides logistic regression model) [14] while a further study [16] revealed no significant association of maternal exposure to GLY with NTDs. No positive associations of parental exposures to GLY with childhood allergies, asthma and bronchitis [19], longer time-to-pregnancy [21, 23], SGA births [20, 24], and pre-term deliveries [20] were reported either. Except for a significant association of pre-conceptional exposure to GLY with late abortions [22], no association of exposure to GLY with gestation losses was found [20, 22]. Overall, except for an excess of risk of ADD/ADHD among the offspring of GLY applicators reported by Garry et al. [18], analytical observational studies reviewed here led to the conclusion that exposures to GLY before conception and/or during pregnancy did not increase the risk of adverse reproductive outcomes.

The lack of association between maternal residential proximity to GLY-spraying areas and NTDs reported by Rull et al (with multiple pesticides logistic regression model) [14] and by Yang et al [16] is not consistent with Carrasco and others’ hypothesis [4, 7] that events taking place during cephalic neural crest development would be the primary target for a putative GLY developmental toxicity. Paganelli et al’s data [4] were obtained on frog and chicken embryos directed exposed to GLY-based herbicides and, as far as the authors are aware, no developmental toxicity study on mammalian species (rodents and lagomorph) has found an increased occurrence of NTDs in GLY exposed fetuses [1].

A major limitation common to all the foregoing studies was an inaccurate assessment of the exposure. In most studies shown in Tables 2 and 3 exposure to the herbicide was analyzed as a dichotomous (exposed / not exposed) variable, and the individual exposure status was inferred from responses to interviews and questionnaires. In one study (Table 3), an ecological-type design was used [23] and thus exposure data was aggregated at the group level as if all individuals living in the studied regions were evenly “exposed” or “not exposed” to GLY.

In fact, the individual exposure to GLY or any other pesticide depends on a number of factors such as wearing protective clothes, the diet, the amount of the pesticide used and others and varies within a population. The exposure or the internal dose received by members of a population is also a continuous variable. The dichotomization of this variable is likely to make analytical studies less sensitive to detect detrimental effects of GLY on health outcomes, if they do occur. For example, a number of individuals assigned to the “exposed group” might have received internal doses of GLY at levels below those (threshold) levels that would lead to adverse outcomes thereby diminishing the power of the study. Moreover, treating exposure as a binary variable precludes a further analysis of dose (concentration) – response relationship when a significant association is eventually found. As highlighted by Hill, a “biological gradient” or a “dose/concentration-response relationship” is one of the aspects to be considered for ruling out possible non-causal explanations for the association [29].

In 4 out of 6 studies evaluating associations between exposure to GLY and the occurrence of birth defects or childhood functional disorders (Table 1), maternal “residential proximity” to GLY sprayed areas was used as a surrogate indicator of exposure to GLY. As far as the authors are aware, no bio-monitoring study has investigated the extent to which “residential proximity” is a valid and reliable non-quantitative indicator of increased exposure to GLY.

There exist only a few bio-monitoring studies on the human exposure to GLY. A bio-monitoring study by Acquavella et al [30] measured the levels of GLY in the 24-h urine samples of 48 farmers, their spouses and children, as part of the Farm Family Exposure Study in Minnesota US. Sixty percent of farmers, 4 % of their spouses and 12 % of their children had detectable levels of GLY in the urine on the day of pesticide application (geometric mean concentration was 3 ppb and the maximum value was 233 ppb). The authors noted that farmers who did not use rubber gloves had urinary levels of GLY higher than the levels found among those who used this protective equipment (10 ppb *versus* 3 ppb). They also observed that all but one child with detectable levels of GLY had helped with the application or were present during pesticide mixing, loading or application [30].

A further study by Curwin et al [31] measured urinary levels of GLY and some other pesticides in farm and non-farm households (47 fathers, 48 mothers and 117 children) living in Iowa (US). Curwin et al’s data demonstrated that detectable levels of GLY are present in the urine of most (>60 %) farm and non-farm US participants [31]. Moreover, detectable levels of GLY have also been found in the urine of farm and non-farm European residents by other bio-monitoring studies [32].

Two more studies published in scientific journals and three unpublished investigations reviewed by Niemann et al [32] also detected GLY in human urine samples. Taken together the seven bio-monitoring studies indicated that amounts of GLY in the ppb (μg/L) range are found in the urine of farm and non-farm individuals in the US and in the EU.

In addition to the foregoing studies on the levels of GLY in urine samples, there is a single study on the blood levels of GLY in pregnant women. Aris and Leblanc [33] measured concentrations of GLY in the blood serum of pregnant (maternal and fetal cord blood) and non-pregnant women from Eastern towns of Quebec, Canada. Participants reported not to have had any direct contact with pesticides and thus the diet remained as the most likely origin of GLY found in their blood. GLY was undetectable (<15 ng/mL) in the maternal and fetal cord blood (*n* = 30), and detected in only 2 out of 39 (5 %) non-pregnant women.

As aforementioned, bio-monitoring studies showed that GLY is detectable in the urine of farm and non-farm individuals. Since soybeans and a variety of other food items may contain trace amounts of GLY, the diet is one of the possible sources of GLY residues found in the urine of farm and non-farm individuals. Occupational exposure through dermal route adds to this background ingestion of GLY residues.

According to Williams et al [1] estimates of exposure for aggregated acute and chronic exposure scenarios based on worst-case assumptions were 125 and 32.3 μg/kg bw/day, respectively. For young children the estimated values were 97 and 52 μg/kg bw/day for acute and chronic exposures, respectively [1]. Based on the worst case assumptions, for an adult female applicator estimated dietary exposure would account for 23.8 μg/kg bw/day while occupational exposure would account for 56.2 (acute) and 8.5 μg//kg bw/day (chronic) exposures. For children aged 1 to 6 years, dietary exposure would account for 51.9 μg/kg bw/d while reentry and bystander exposures and some infrequent events would account for the additional exposure [1]. It is of note that for occupational exposure of applicators, according to Williams et al [1], the highest measured value from all biomonitoring studies was used for the “worst case” estimation of acute exposure. Since as a rule the monitoring studies were conducted on developed countries, the question arises as to whether the highest estimated values could be even higher in developing country scenarios where farmers do not routinely wear protective clothes and equipment.

Acquavella et al’s study [31], for instance, demonstrated that, among farmers, the highest urinary levels of GLY were recorded on the day of application, and when protective gloves were not used during mixing, loading or spraying the herbicide. According to the authors, only 5 % of farmers’ spouses and 12 % of their children had detectable levels of GLY in the urine, and all but one of the contaminated children had reported a direct contact with the herbicide product [31]. These findings raise doubts whether a mere residential proximity to cultivated areas does in fact add significantly to the background exposure to GLY that occurs through the diet irrespective of the individual place of residence.

## Conclusions

In summary, except for a possible association with Attention Deficit Hyperactivity Disorder needing confirmation by further studies – data from existing epidemiologic studies do not lend support to the notion that GLY is a human reproductive and developmental toxicant. Nonetheless, this set of predominantly negative findings is far from providing a definitive evidence that occupational exposure to GLY poses no risk for human development and reproductive health. This systematic review found only a few studies on people occupationally exposed to GLY and, as a rule, the existing studies used non-quantitative estimations of exposure. Moreover, investigations on the potential adverse effects of GLY identified in the systematic review are heterogeneous regarding the developmental and reproductive outcomes studied. The variability in the outcomes investigated (e.g., one study on hypospadias, gastroschisis, and oral clefts, and two studies on NTDs) limits the evaluation of the consistency of specific negative findings across epidemiologic studies. Finally, there is no analytical observational study conducted on developing countries where farmers are exposed or likely to be exposed to higher internal doses of GLY because in tropical regions not to use routinely protective clothes and equipment when mixing, loading and spraying pesticides is the rule rather than an exception [34]. Along this line, human risk assessment would greatly benefit from a set of good quality epidemiologic studies, particularly from prospective cohort studies with quantitative estimations of exposure (e.g. by measuring urinary levels of GLY) before pre-conception and during pregnancy.

## Abbreviations

ADD/ADHD, attention deficit disorder / attention deficit hyperactivity disorder; AHS, agricultural health study; AMPA, aminomethylphosphonic acid; ANVISA, National agency for sanitary surveillance – Brazil; BIREME/BVS, Regional library of medicine/ health virtual library – Brazil; CFR, conditional fecundability ratio; ECLAMC, Latin American collaborative study on congenital malformations; EPSPS, enolpyruvylshikimate phosphate synthase; GLY, glyphosate; GM, genetically modified; IARC, International Agency for Research on Cancer; LILACS, Latin American and the Caribbean Literature on Health Sciences; MRCs, maximum residue concentrations; NTDs, neural tube defects; OFFHS, Ontario Farm Family Health Study; SGA, small-for-the gestational-age birth
